# Limonene enhances rice plant resistance to a piercing‐sucking herbivore and rice pathogens

**DOI:** 10.1111/pbi.14481

**Published:** 2024-09-28

**Authors:** Chang‐Lai Qiu, Wei Li, Ling‐Nan Wang, Shi‐Cheng Wang, Supaporn Falert, Chao Wang, Shi‐Yu Yu, Sara Taha Abdelkhalek, Jing Lu, Yong‐Jun Lin, Man‐Qun Wang

**Affiliations:** ^1^ College of Plant Science and Technology Huazhong Agricultural University Wuhan China; ^2^ National Key Laboratory of Crop Genetic Improvement and National Center of Plant Gene Research Huazhong Agricultural University Wuhan China; ^3^ Department of Entomology, Faculty of Science Ain Shams University Cairo 11566 Egypt; ^4^ Institute of Insect Sciences Zhejiang University Hangzhou 310058 China

**Keywords:** *Oryza sativa*, herbivore‐induced plant volatiles, limonene, limonene synthases, brown planthopper, phytopathogen resistance

## Abstract

Terpene synthases (TPSs) are key enzymes in terpenoids synthesis of plants and play crucial roles in regulating plant defence against pests and diseases. Here, we report the functional characterization of OsTPS19 and OsTPS20, which were upregulated by the attack of brown planthopper (BPH). BPH female adults performed concentration‐dependent behavioural responses to (*S*)‐limonene showing preference behaviour at low concentrations and avoidance behaviour at high concentrations. Overexpression lines of *OsTPS19* and *OsTPS20*, which emitted higher amounts of the monoterpene (*S*)‐limonene, decreased the hatching rate of BPH eggs, reduced the lesion length of sheath blight caused by *Rhizoctonia solani* and bacterial blight caused by *Xanthomonas oryzae*. While knockout lines of *OsTPS19* and *OsTPS20*, which emitted lower amounts of (*S*)‐limonene, were more susceptible to these pathogens. Overexpression of *OsTPS19* and *OsTPS20* in rice plants had adverse effects on the incidence of BPH, rice blast, and sheath blight in the field and had no significant impacts on rice yield traits. OsTPS19 and OsTPS20 were found to be involved in fine‐tuning the emission of (*S*)‐limonene in rice plants and play an important role in defence against both BPH and rice pathogens.

## Introduction

As immobile organisms, plants have evolved comprehensive and effective defence strategies to counter attacks from herbivores and phytopathogens. These mechanisms include signal perception of cues from disease and pest invasion, transduction of phytohormone signals, and synthesis of defensive secondary metabolites and enzymes (Erb and Reymond, 2019; Mithöfer and Boland, 2012; Ngou *et al*., 2022). Among the secondary metabolites, plant volatile organic compounds (VOCs) are released to convey information to the surrounding environment (Dudareva *et al*., 2013; Sharifi *et al*., 2018). Herbivore‐induced plant volatiles (HIPVs) benefit plants by recruiting parasitoids and predators for indirect defence, which is known as the “cry for help” strategy (Aartsma *et al*., 2017; Kessler and Baldwin, 2001; Turlings and Erb, 2018). HIPVs, regulate plant–herbivore relationships (Jin *et al*., 2023; Lin *et al*., 2021; Liu *et al*., 2021), trigger defensive responses of surrounding healthy plants (Gong *et al*., 2023; Jing *et al*., 2021a, 2021b), and directly destroy herbivores by increasing toxicity (Chen *et al*., 2021; Maurya *et al*., 2020; Veyrat *et al*., 2016). Moreover, VOCs induced by pathogenic microorganisms, especially terpenes, benefit plants by disrupting the lifestyle of harmful microorganisms (Hammerbacher *et al*., 2019; Ma *et al*., 2022; Xu *et al*., 2021b), influencing host immunity (Jin *et al*., 2023; Riedlmeier *et al*., 2017; Song and Ryu, 2018), and prompting plants to recruit beneficial microorganisms (Kong *et al*., 2021; Liu and Brettell, 2019; Sharifi *et al*., 2022). Such regulated VOCs can affect the interactions between plants, herbivores, and phytopathogens (Eberl *et al*., 2018, 2020; Liu *et al*., 2022).

The brown planthopper (BPH), *Nilaparvata lugens*, a piercing‐sucking pest of rice, has severe deleterious effects on rice yield by feeding on phloem, ovipositing in the leaf sheath, and transmitting viruses (Heong *et al*., 2015). BPH‐damaged, rice plants emit large amounts of volatile compounds with varying ecological functions. Compounds, such as DMNT, (*E*)‐*β*‐caryophyllene, linalool, methyl salicylate, (*Z*)‐3‐hexenal, and (*E*)‐2‐hexenal attract *Anagrus nilaparvatae* (Hu *et al*., 2020; Mao *et al*., 2018; Xiao *et al*., 2012), while 2‐heptanone and 2‐heptanol caryophyllene oxide and linalool repel BPH (Duan *et al*., 2022; Lu *et al*., 2014; Xiao *et al*., 2012). Under BPH attack, limonene emission increased (Lou *et al*., 2006; Xiao *et al*., 2012), and limonene synthases were dramatically upregulated (Xu *et al*., 2021a). Limonene is known for its ability to inhibit several microorganisms, including *Staphylococcus aureus* (Celaya *et al*., 2014), *Listeria monocytogenes* (Han *et al*., 2019), *Candida* sp. (D'Arrigo *et al*., 2019), *Bacillus subtilis* and *Escherichia coli* (Fahim *et al*., 2019), and tobacco mosaic virus (Luo *et al*., 2023).

Terpene synthases (TPSs) generate volatile terpenoids (monoterpenoids, sesquiterpenoids, and homoterpenes), via the mevalonate (MVA) and the methyerythritol (MEP) pathways (Abbas *et al*., 2017; Chen *et al*., 2011). Some TPSs have been identified and used in the manipulation of VOCs to improve the ability of plants to control pests and diseases. For instance, the transformation of *PlTPS3* and *PlTPS4* from lima bean into rice results in the recruitment of *Cotesia chilonis*, parasitic wasps of the striped rice stemborer, due to the enhanced production of (*E*)‐4,8‐dimethyl‐1,3,7‐nonatriene (DMNT) and (*S*)‐linalool (Li *et al*., 2018, 2021). Linalool and caryophyllene, catalysed by *OsTPS3* and *OsTPS29*, respectively, suppressed the growth of the fungus *Magnaporthe oryzae* (Wang *et al*., 2023). *OsTPS19* (*Os04g27190*) and *OsTPS20* (*Os04g27340*), two duplicated defence TPSs genes, were significantly induced in rice plants in response to BPH infestation. In rice plants, OsTPS19 functions as an (*S*)‐limonene synthase in rice plants and its overexpression boosts against *M. oryzae* (Chen *et al*., 2018). OsTPS20 is activated by oxidative stress or bacterial infection by *Xanthomonas oryzae pv. oryzae* (*Xoo*), and its recombinant protein generates a variety of monoterpenes including (*S*)‐limonene (Lee *et al*., 2015, 2016).

By generating knockout and overexpression lines of *OsTPS19* and *OsTPS20*, we investigated the regulatory and defensive roles of limonene against BPH and diseases in rice. The biological functions of (*S*)‐limonene were investigated by preference and performance of BPH and pathogen incidence in wild‐type and transgenic plants. In addition, odour preference and direct toxicity to eggs during incubation were examined using synthetic (*S*)‐limonene. In the field trials, herbivore resistance, disease resistance, and agronomic trials were evaluated. The results show that boosting the release of (*S*)‐limonene can potentially improve resistance to BPH and pathogens.

## Results

### Effects of female BPH infestation on rice plant volatiles

**Figure 1:**
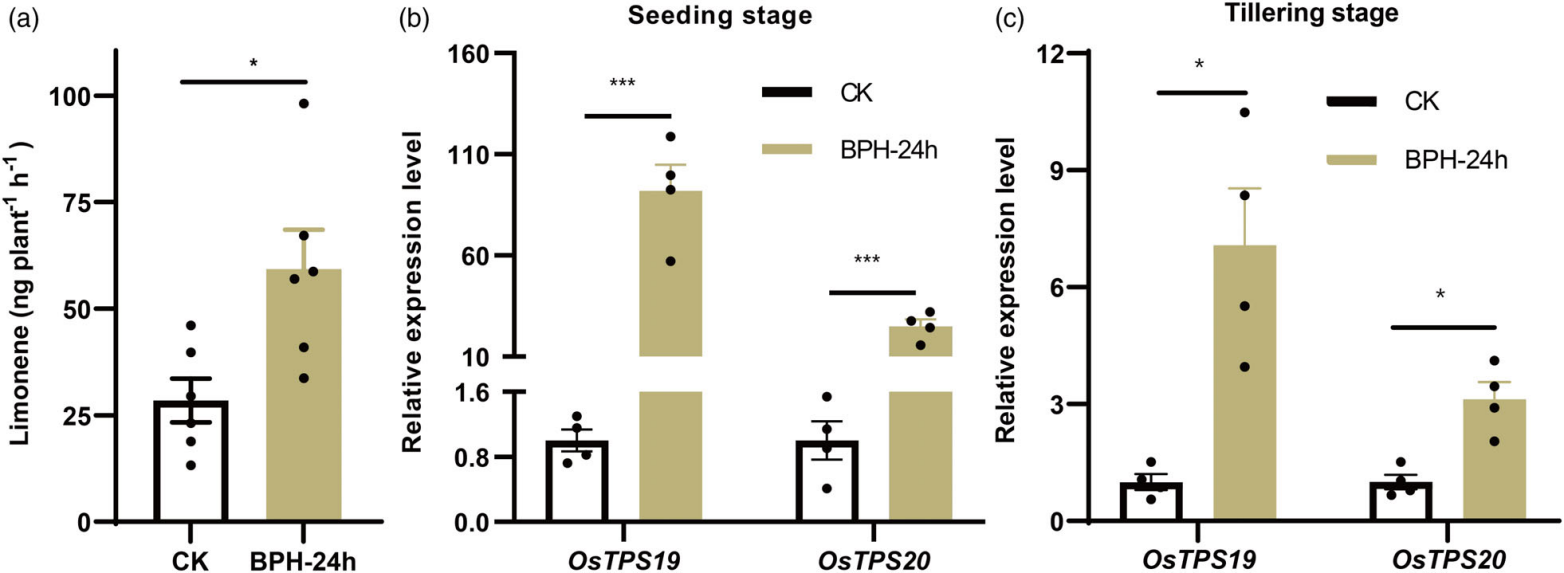
BPH infestation increased limonene emissions and limonene synthases transcription. (a) Limonene emission from ZH11 infested with female adults for 24 h or not, *n* = 6. (b, c) Relative expression level of *OsTPS19* and *OsTPS20* transcription from healthy or BPH‐infested sheath at seeding stage and tillering stage, rice were 20 days old and 40 days old respectively, *n* = 4, seeding stage: 20 days after planting, tillering stage: 40 days after planting, CK: healthy ZH11, BPH‐24 h: ZH11 infested with 10 gravid female adults for 24 h. Values presented are the means ± SE. Asterisks indicate statistically significant differences, Student's *t*‐tests, *P < 0.05, ***P < 0.001.

Absolute quantification of (S)‐limonene was calculated using a standard curve (Figure S1). BPH infestation significantly affected the release of (*S*)‐limonene in rice plants. The level of (*S*)‐limonene emitted by rice plants infested with 10 gravid BPH females for 24 h was 2.08 times higher than that of healthy plants (Figure 1a). After gravid BPH infestation, the emission levels of linalool, *α*‐bisabolene, and cedrol increased (Figure S2). The relative expression of *OsTPS19* and *OsTPS20* in seedling stage rice plants elevated by 62.02 and 24.94 times, respectively (Figure 1b), while those in tillering stage rice plants increased by 7.08 and 3.13 times, respectively, in BPH‐infested plants (Figure 1c). Furthermore, *OsTPS19* had elevated transcription levels in the bud during the embryonic stage, in the sheath and leaf during the seeding stage and sheath during the tillering stage. On the other hand, *OsTPS20* exhibited increased transcription levels in the roots during the seeding stage, in leaves during the tillering stage, and in spikes during the spiking stage (Figure S3).

### Effects of altered expression *OsTPS19* and *OsTPS20* on rice plant volatiles

**Figure 2:**
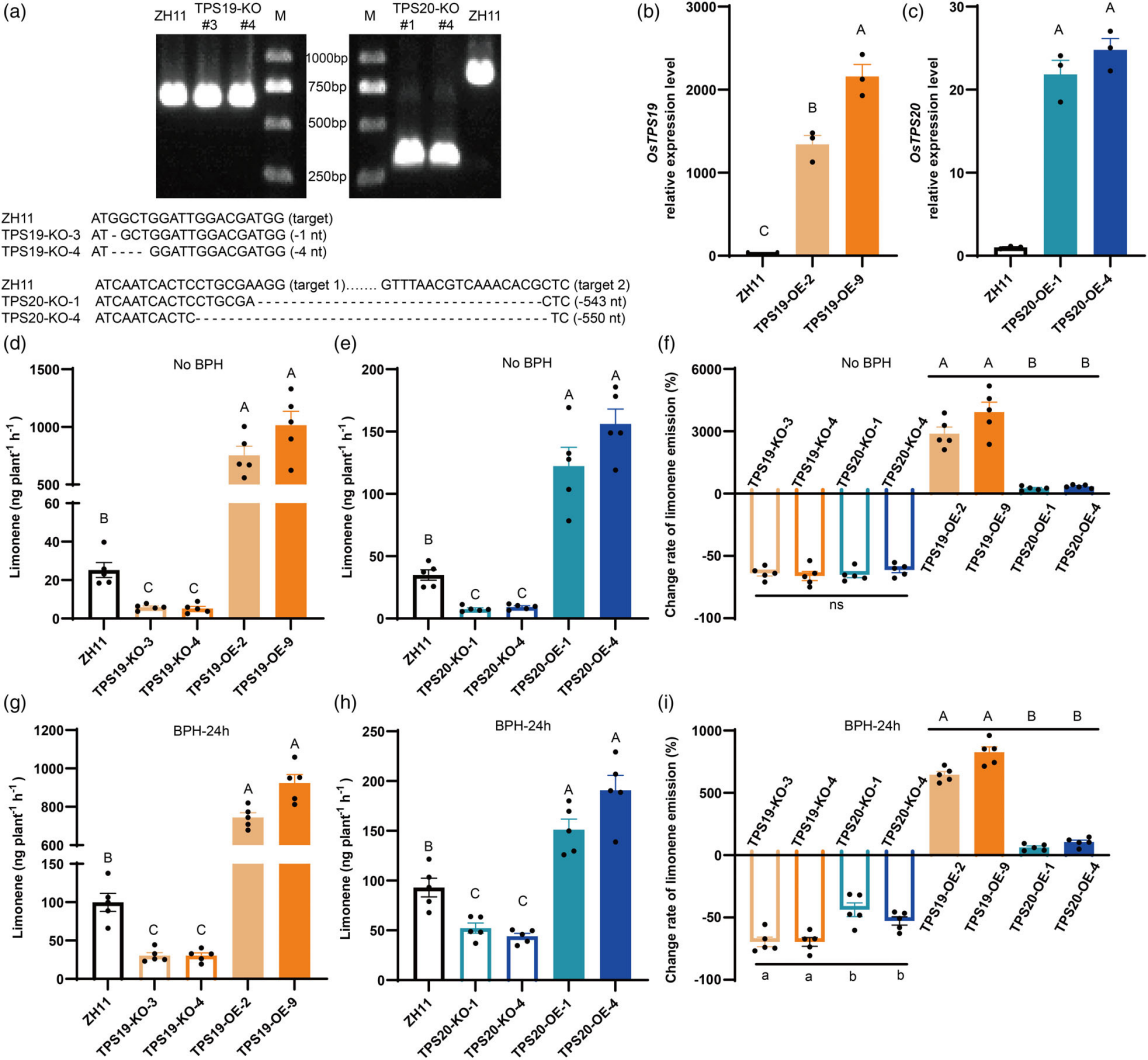
Limonene emissions reduced in knockout lines and increased in overexpression lines. (a) Sequencing of *OsTPS19* and *OsTPS20* knockout lines for homozygote. (b, c) *OsTPS19* and *OsTPS20* transcription in overexpression lines, *n* = 3. (d, e, f) The limonene emission and change rate relative to ZH11 (%) from healthy rice plants, *n* = 5, No BPH: non‐infested with BPH. (g, h, i) The limonene emission and change rate relative to ZH11 from rice plants after BPH infestation, *n* = 5, BPH‐24 h: infested with 10 gravid female adults for 24 h. Values presented are the means ± SE. Different capital letters indicate extremely significant differences (*P* < 0.01), different lowercase letters indicate significant differences (*P* < 0.05), ordinary one‐way ANOVAs with Tukey's HSD test.

*Agrobacterium tumefaciens*‐mediated transformation was used to generate knockout and overexpression lines of *OsTPS19* and *OsTPS20*. Sequencing results indicated that TPS19‐KO‐3 and TPS19‐KO‐4 had deletions of 1 nt and 4 nt, resulting in a frameshift mutation, while TPS20‐KO‐1 and TPS20‐KO‐4 displayed a substantial fragment deletion between two gRNA target sites (Figure 2a; Figure S4). Southern blot analysis showed that TPS19‐OE‐2, TPS19‐OE‐9, TPS20‐OE‐1, and TPS20‐OE‐4 were single‐copy lines (Figure S5). Compared with the wild‐type, the expression levels of *OsTPS19* were significantly increased 1342 times in TPS19‐OE‐2 and 2159 times in TPS19‐OE‐9 (Figure 2b). For *OsTPS20*, the expression levels were significantly increased 21.84 times in TPS20‐OE‐1 and 24.78 times in TPS20‐OE‐4 (Figure 2c).

Compared with the wild‐type, the emission of (*S*)‐limonene increased in the overexpression lines and decreased in the knockout lines (Figure 2d, e). Relative to the wild‐type, the growth rates of limonene release were higher in *OsTPS19* than those in *OsTPS20* overexpression lines (Figure 2f). After BPH infestation, the emission of (*S*)‐limonene also increased in the overexpression lines and decreased in the knockout lines (Figure 2g, h), the change rates of limonene release were higher in *OsTPS19* overexpression lines and knockout lines (Figure 2i). In addition, the relative amount of linalool and *β*‐myrcene were significantly decreased in *OsTPS19* overexpression lines, while these differences disappeared after BPH infestation (Figure S6a). Overexpression of *OsTPS20* had no effect on the emission of other volatiles (Figure S6b).

### (*S*)‐limonene influenced oviposition preference and performance of BPH female adults

**Figure 3:**
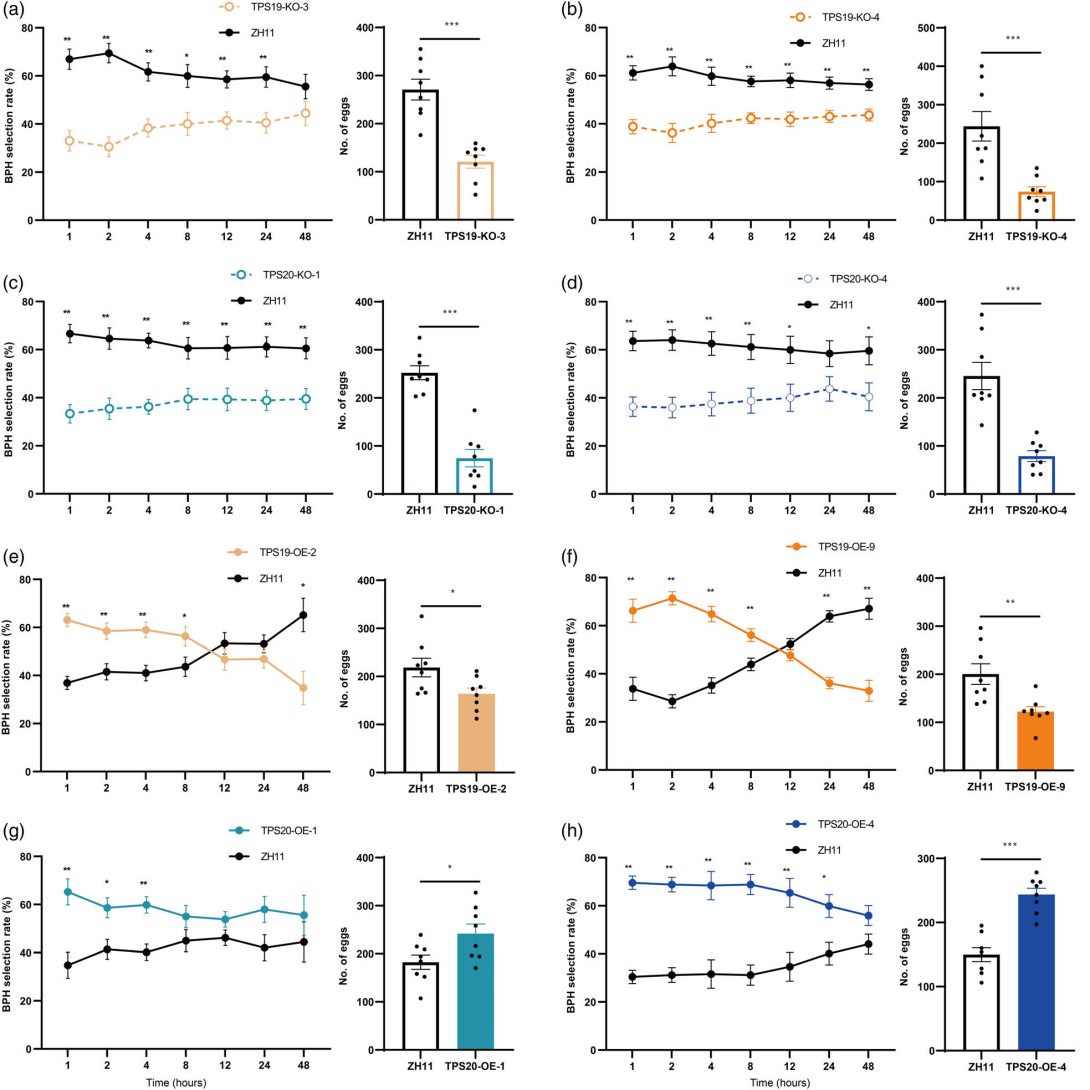
Transgenic rice plants affected the oviposition preference of BPH female adults. BPH select rate (%) and number of laid eggs within 48 h between ZH11 and TPS19‐KO‐3 (a), TPS19‐KO‐4 (b), TPS20‐KO‐1 (c), TPS20‐KO‐4 (d), TPS19‐OE‐2 (e), TPS19‐OE‐9 (f), TPS20‐OE‐1 (g), and TPS20‐OE‐4 (h). Experiments were repeated eight times, dots represent biological replicates, and values presented are the means ± SE. Asterisks indicate statistically significant differences, Student's *t*‐tests, *P < 0.05, **P < 0.01, ***P < 0.001.

**Figure 4:**
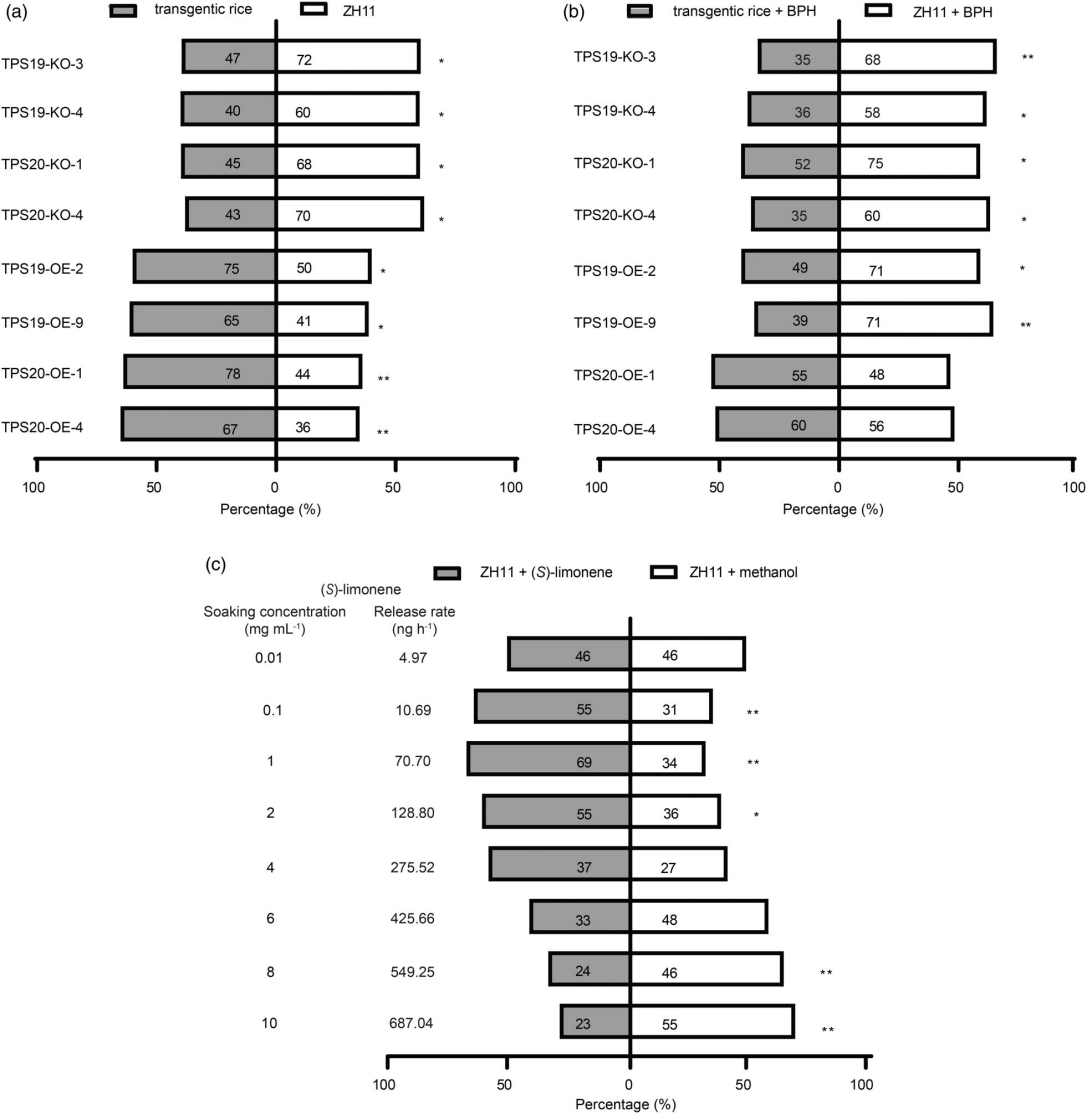
(*S*)‐limonene affected host preference of BPH female adults. (a) Choice percentage (%) of female adults to transgenic lines and ZH11. (b) Choice percentage (%) of female adults to transgenic lines and ZH11 infested with 10 gravid female adults for 24 h. (c) Choice percentage (%) of female adults to ZH11 with (*S*)‐limonene or methanol, the numbers on left represent the soaking concentrations and release rates of (*S*)‐limonene. Experiments were repeated eight times, all 160 female adults. The numbers on each bar represent the total number of female adults which chose the odour source. Asterisks indicate statistically significant differences, χ‐squared test, *P < 0.05, **P < 0.01.

The oviposition preference of BPH female adults was evaluated using choice experiments. In 48 h paired choice experiments with rice, gravid BPH adult females showed an avoidance behaviour to knockout lines of *OsTPS19* and *OsTPS20* (Figure 3a–d), while they showed preference behaviour to overexpression lines of *TPS20* (Figure 3g, h). Notably, gravid adult females showed a preference behaviour for overexpression lines of *TPS19* at the initial phase of the experiment but exhibited an avoidance behaviour at the end of the experiment (Figure 3e, f). Similar results were observed in H‐tube olfactometer assays. BPH showed an avoidance behaviour towards the knockout lines of *OsTPS19* and *OsTPS20*, whereas they showed preference behaviour to overexpression lines of *TPS19* and *TPS20* (Figure 4a). After 10 gravid adult females infested a plant for 24 h, BPH still showed avoidance behaviour to the knockout lines of *OsTPS19* and *OsTPS20*, they showed avoidance behaviour and no preference to the overexpression lines of *OsTPS19* and *OsTPS20* (Figure 4b). BPH displayed a dose‐dependent response behaviour to synthetic (*S*)‐limonene, showing a preference for (*S*)‐limonene at a release rate of 4.97–128.80 ng h^−1^, and an aversion to it at a release rate from 549.25 to 687.04 ng h^−1^ (Figure 4c).

**Figure 5:**
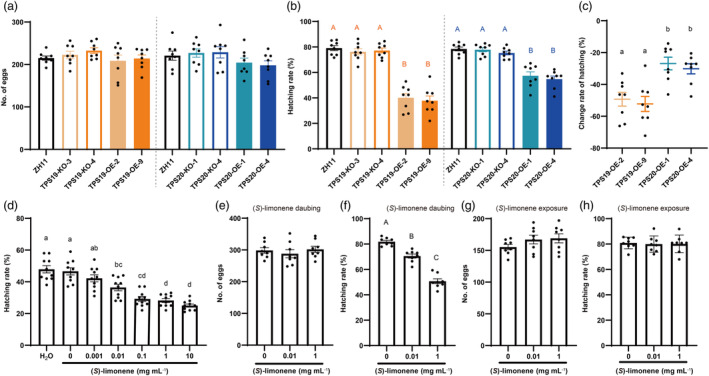
(*S*)‐limonene decreased hatching rate of BPH eggs. (a–c) Total numbers, hatching rate (%), and change rate relative to ZH11 (%) of BPH eggs on transgenic lines or ZH11 that were individually infested by two pairs of BPH adults for 7 days, *n* = 8. (d) Hatching rate (%) of separated eggs with H_2_O, lanolin or (*S*)‐limonene incubating, *n* = 10. (e, f) Total numbers and hatching rate (%) of BPH eggs on ZH11 that were individually infested with 10 BPH gravid female adults for 24 h after lanolin or (*S*)‐limonene daubing, *n* = 8. (g, h) Total numbers and hatching rate (%) of BPH eggs on ZH11 that were individually infested by 10 BPH gravid female adults for 24 h after lanolin or (*S*)‐limonene exposing, *n* = 8. Values presented are the means ± SE, dots represent biological replicates. Different lowercase letters indicate statistically significant differences (*P* < 0.05) and different capital letters indicate extremely significant differences (*P* < 0.01), ordinary one‐way ANOVAs with Tukey's HSD test.

While altered expression of *OsTPS19* and *OsTPS20* had no impact on the oviposition performance of BPH female adults the total number of eggs deposited remained constant rice genotypes (Figure 5a, b), the hatching rate of eggs laid on overexpression lines of *OsTPS19* and *OsTPS20* was diminished compared with other genotypes (Figure 5b, d). Relative to the wild‐type, the decline in hatching rate was higher in *OsTPS19* than in *OsTPS20* overexpression lines (Figure 5c). The hatching rate of eggs decreased with the increasing (*S*)‐limonene concentration, as shown in Figure 5d (eggs incubated in flat plates). The 0.01 and 1 mg mL^−1^ concentrations of synthetic (*S*)‐limonene had no effect on BPH oviposition (Figure 5e), but reduced the hatching rate of eggs (Figure 5f). Exposure to (*S*)‐limonene at concentrations of 0.01 and 1 mg mL^−1^ did not affect the number of eggs and hatching rate of eggs in adjacent plants (Figure 5g, h). (*S*)‐limonene had direct toxicity effects on BPH eggs.

In TPS19‐OE‐9 lines, the developmental time from nymph to adult BPH females was longer than in *OsTPS19* knockout lines (Figure S7a), but there were no significant differences in adult weight (Figure S7e–h), longevity (Figure S7i–l), or nymph survival (Figure S7m, n) among the different genotypes. (*S*)‐Limonene had slight effects on the development of BPH.

**Figure 6:**
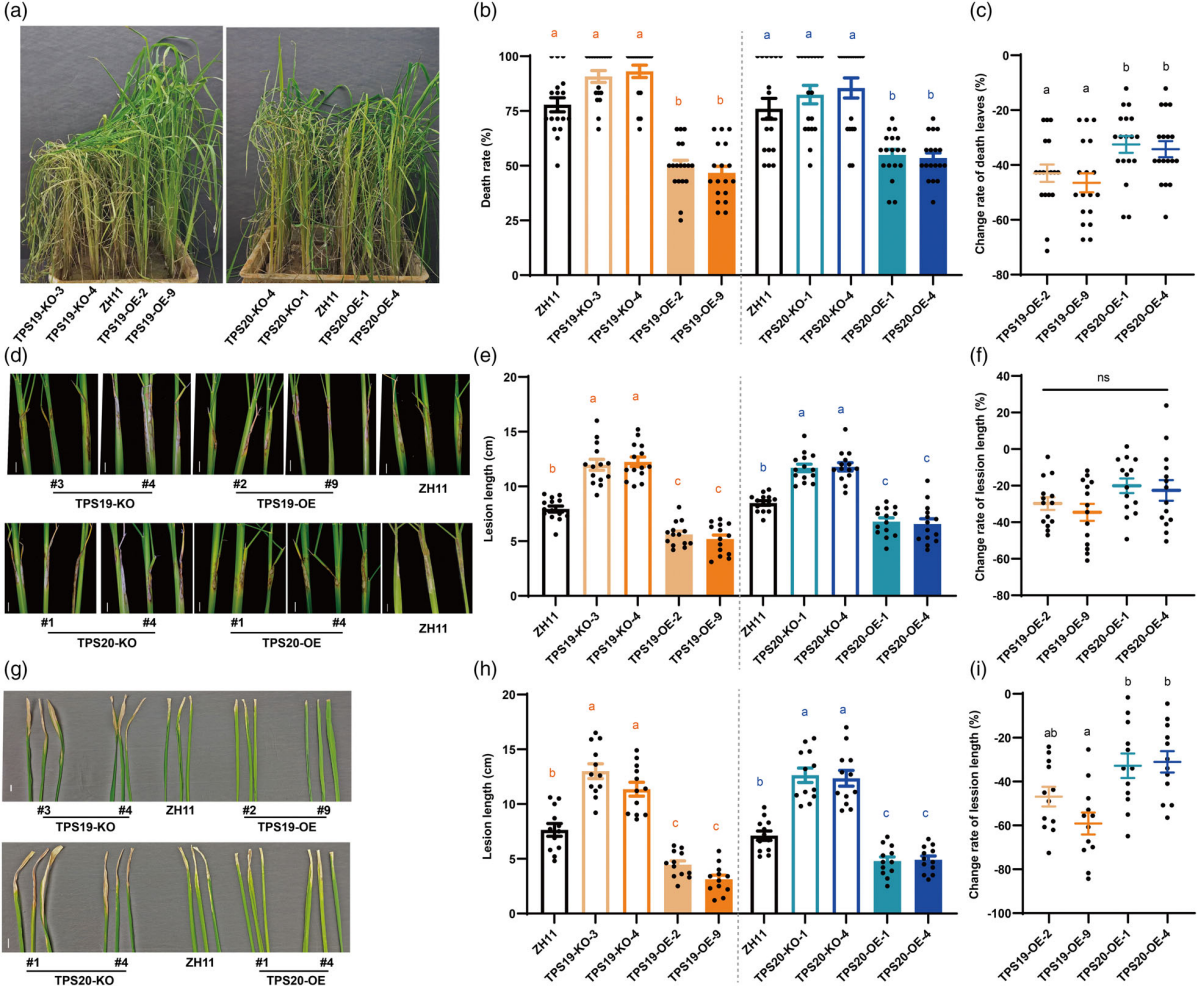
Overexpression of *OsTPS19* and *OsTPS20* enhanced resistance against BPH and pathogenic microorganisms under laboratory conditions. (a) Damage phenotypes of transgenic lines and ZH11 that were infested with 450 BPH female adults for 18 days. (b, c) Leaf death rate and its change rate on transgenic lines and ZH11 that were infested with 450 BPH female adults for 18 days, *n* = 3 × 6. (d) Damage phenotypes of transgenic lines and ZH11 infested by *R.solani* at 7 dpi, bar indicates 2 cm. (e, f) Lesion length and its change rate on transgenic lines and ZH11 infested by *R.solani* at 7 dpi, *n* = 14. (g) Damage phenotypes of transgenic lines and ZH11 infested by *Xoo* at 14 dpi, bar indicates 2 cm. (h, i) Lesion length and its change rate on transgenic lines and ZH11 infested by *Xoo* at 14 dpi. Values presented are the means ± SE, and dots represent biological replicates. Different lowercase letters indicate statistically significant differences (*P* < 0.05), Kruskal–Wallis test with Dunn's test in (b, c) and ordinary one‐way ANOVAs with Tukey's HSD test in (e, f, h, i).

To better mimic the field environmental conditions, numerous BPH adults were introduced in cage experiments. Compared with the wild‐type, the leaf death rate was lower in *TPS19* and *TPS20* overexpression lines, while the leaf death rate was slightly higher in the *TPS19* and *TPS20* knockout lines (Figure 6a, b). Compared with the wild‐type and knockout lines, the decline rates of dead leaves in *OsTPS19* were higher than those in *OsTPS20* overexpression lines (Figure 6c).

### (*S*)‐limonene inhibits rice pathogenic microorganisms

Based on a prior research that (*S*)‐limonene increased rice resistance to *M. oryzae* (Chen *et al*., 2018), this study investigated the effect of (*S*)‐limonene on *Rhizoctonia solani* and *Xoo* infection under laboratory conditions. In the overexpression lines of *TPS19* and *TPS20*, the lesion length was shorter after inoculation with *R. solani* or *Xoo* compared with wild‐type and knockout lines (Figure 6e, h). Relative to the wild‐type, there were no differences between *OsTPS19* and *OsTPS20* overexpression lines in the reduction rates of *R. solani* lesion length (Figure 6f), while the decline rates of *Xoo* lesion length in TPS19‐OE‐9 lines were higher than those in *OsTPS20* overexpression lines (Figure 6i). Overexpression of *TPS19* and *TPS20* in rice plants enhanced resistance to *R. solani* and *Xoo*, while *TPS19* and *TPS20* knockout lines were more susceptible to the pathogens.

### Ecological effects of (*S*)‐limonene in the field

**Figure 7:**
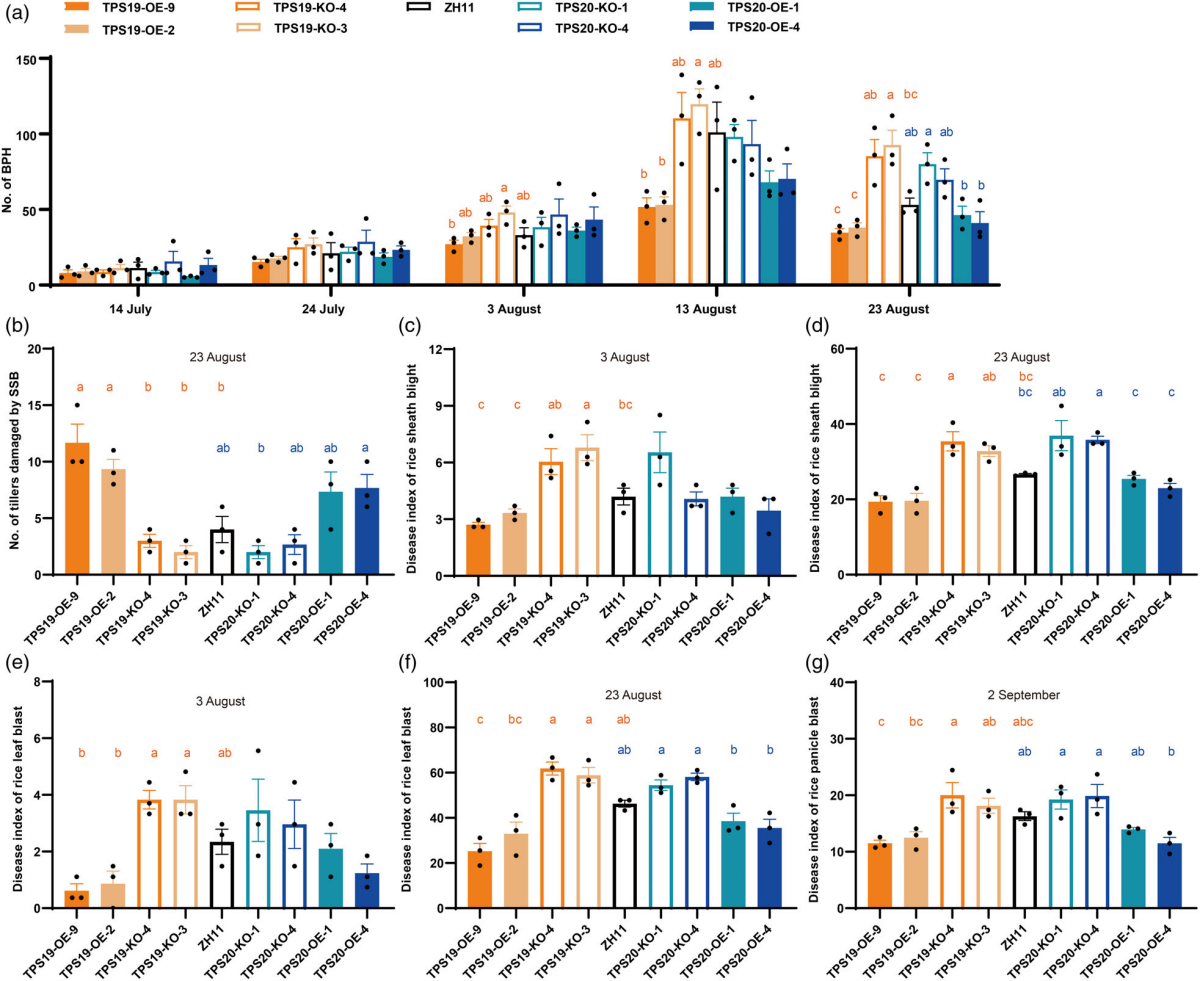
Overexpression of *OsTPS19* and *OsTPS20* reduced the population of BPH and the incidence of pathogenic microorganisms in the field. (a) Number of BPH at 14 July, 24 July, 3 August, 13 August and 23 August, *n* = 3. (b) Number of tillers damaged by SSB at 23 August, *n* = 3. (c, d) Disease index of rice sheath blight at 3 August and 23 August, *n* = 3. (e, f) Disease index of rice leaf blast at 3 August and 23 August, *n* = 3. (g) Disease index of rice panicle blast at 2 September, *n* = 3. Values presented are the means ± SE, dots represent biological replicates. Different orange lowercase letters indicate statistically significant differences (*P* < 0.05) between ZH11 and transgenic lines of *OsTPS19*, different blue lowercase letters indicate statistically significant differences (*P* < 0.05) between ZH11 and transgenic lines of *OsTPS20*, ordinary one‐way ANOVAs with Tukey's HSD test.

The increased amount of (*S*)‐limonene in rice plants inhibited BPH development while promoting the occurrence of striped stem borer (*Chilo suppressalis*, SSB). As shown in Figure 7a, TPS19‐OE and TPS20‐OE lines had less BPH than their respective KO counterparts (TPS19‐KO and TPS20‐KO‐1). SBB‐induced damage to tillers was more significant in TPS19‐OE than in both wild‐type and TPS19‐KO lines, whereas damage was more prominent in TPS20‐OE‐4 compared with TPS20‐KO‐1 lines (Figure 7b). No significant differences were observed in the rice leaf roller (*Cnaphalocrocis medinalis*, RLR) damage rates (Figure S8a, b) or tiller damage rates (Figure S8c) caused by SSB between various rice lines on August 3.

The enhanced (*S*)‐limonene content in rice plants reduced the incidence of rice blast and sheath blight. The disease indices of leaf blast and sheath blight were lower in TPS19‐OE and TPS20‐OE lines compared with TPS19‐KO and TPS20‐KO lines, respectively (Figure 7c–g). While overexpression of TPSs did not affect the yield traits of rice plants, TPSs knockouts reduced them. There were no significant variations in height among various genotypes (Figure S9b), and both TPS19‐KO and TPS20‐KO lines had fewer panicles (Figure S9c) and lighter seeds (Figure S9d).

## Discussion

Although the biological functions of BPH‐derived rice volatiles have been extensively studied, the role of (*S*)‐limonene, specifically those induced by BPH, has received scant attention. Based on the higher emission of (*S*)‐limonene in healthy rice (compared with other volatiles) (Lou *et al*., 2006; Sun *et al*., 2021; Xiao *et al*., 2012) and its association with herbivores infestation (Lin *et al*., 2008; Moreira *et al*., 2018; Yuan *et al*., 2008), we revealed the direct defensive role of limonene against BPH in rice plants.

After BPH infestation, both the expression of *OsTPS19* and *OsTPS20* (limonene synthases genes) and the release of limonene were observed to increase (Figure 1), as previously reported by Xiao *et al*. (2012) and Xu *et al*. (2021a). These results showed that the release of limonene was minimal in the absence of BPH infestation (Figure 2d, e) and increased after BPH infestation in TPS19‐KO or TPS20‐KO lines (Figure 2g, h), suggesting that *OsTPS19* and *OsTPS20* share functional redundancy. Under BPH infestation, the decline rates of limonene release in *OsTPS19* overexpression lines were higher than those in *OsTPS20* overexpression lines (Figure 2i), suggesting that *OsTPS19* may play a more important role in the interaction between rice and BPH. Distinct tissue expression profiles in healthy rice and induction of *OsTPS20* by abiotic factors (Lee *et al*., 2015) indicated that *OsTPS19* and *OsTPS20* might exhibit distinct regulatory and response patterns.

Limonene can repel (Hieu *et al*., 2010; Ling *et al*., 2023; Showler *et al*., 2019) or attract (Li *et al*., 2019; Mérida‐Torres *et al*., 2023; Togashi *et al*., 2019) several arthropod species, depending on the context. Our findings revealed that BPH showed preference behaviour towards (*S*)‐limonene in the low concentration range, while it showed avoidance behaviour towards (*S*)‐limonene in the high concentration range (Figure 4c). Similar results were reported that *Tetranychus urticae* exhibited preference behaviour to low‐dose and showed avoidance behaviour to high‐dose of limonene (Agut *et al*., 2015; Mérida‐Torres *et al*., 2023). The preference shift caused by the concentration‐dependent effect of limonene corresponds to the “preference‐performance” principle. Previous research revealed that an adequate nitrogen supply to rice promoted the release of limonene, rendering it more attractive to BPH and enhanced the accessibility of nutrients for BPH development and reproduction of BPH (Sun *et al*., 2021). BPH avoids rice plants with high limonene emissions to prevent lower egg‐hatching rates. In addition, previous studies have revealed the phenomenon of mutual benefit between BPH and SSB (Hu *et al*., 2020; Wang *et al*., 2018). Particularly, mature SSB females show attractive behaviour to volatiles from rice with BPH pre‐infestation (Liu *et al*., 2021). More tillers were damaged by SSB in TPS19‐OE and TPS20‐OE lines, suggesting the potential significance of (*S*)‐limonene as a semiochemical in this interspecies interaction (Figure 7b). Our results indicated that (*S*)‐limonene is a chemical cue for BPH and SSB to find a more suitable host, so additional research is necessary to understand the ecological and evolutionary significance of (*S*)‐limonene in plant–herbivore interactions.

Interestingly, BPH adults showed a preference for TPS19‐OE lines with non‐BPH infestation (Figure 4a) but showed avoidance behaviour towards TPS19‐OE lines after BPH infestation (Figures 3e, f and 4b). The observed phenomenon could not be explained by the variations in (*S*)‐limonene alone because (*S*)‐limonene at levels similar to those observed in TPS19‐OE lines repelled BPH in synthetic (*S*)‐limonene choice assays (Figures 2d, g and 4c). It was noticed that the relative amount of linalool and *β*‐myrcene significantly decreased in TPS19‐OE lines, while the observed decrease terminated after BPH infestation (Figure S6a). In TPS20‐OE lines, the relative amount of linalool and *β*‐myrcene had a downward trend with no significant differences (Figure S6b). While linalool showed a repellent effect on BPH (Duan *et al*., 2022, 2023; Xiao *et al*., 2012), we hypothesized that it was low amounts of linalool in healthy TPS19‐OE lines explained the BPH preference behaviour. The reduction of some monoterpenes was caused by monoterpenes sharing the same precursor substance geranyl pyrophosphate (GPP) (Dudareva *et al*., 2013; Nagegowda and Gupta, 2020; Yu and Utsumi, 2009). With an increase in substrates, monoterpenes were recovered from lower levels and TPS expression increased after herbivore attack (He *et al*., 2022; Xiao *et al*., 2012; Xu *et al*., 2021a). Therefore, more emphasis needs to be placed on assessing the overall effects of volatiles in complex environments and the alterations in unintended metabolites caused by crosstalk in metabolic networks.

Limonene displays contact and ovicidal toxicity against a variety of organisms (de Mello *et al*., 2023; Showler *et al*., 2019). In our study, (*S*)‐limonene exhibited ovicidal activity against BPH, reducing hatching (Figure 5) while minimally affecting growth (Figure S7). Although the precise mechanism by which limonene exerts its toxic effects is still not fully elucidated, limonene disrupts normal epidermal structure (de Mello *et al*., 2023) and inhibits acetylcholinesterase activity (Liao *et al*., 2022). Instead of feeding on mesophyll tissue, BPH, as a piercing‐sucking pest, feeds on phloem sap, which escapes from the contact toxicity effect of limonene. Nevertheless, BPH eggs are deposited in the leaf sheath, and limonene in rice glands may degrade the epidermis of eggs in the sheath, resulting in fewer nymphs hatching. The combined effects resulted in increased survival of rice in the laboratory (Figures 6a, b and 7a) and reduced the BPH population on overexpression lines in the field (Figure 7a). Due to higher (*S*)limonene emissions, the effects of suppressing BPH egg hatching (Figure 5c) and improving rice plants survival (Figure 6c) were stronger in *OsTPS19* overexpression lines. Future research is required to elucidate the precise mechanism by which limonene affects embryo integrity.

Limonene possesses a broad spectrum of microbiological activity, including *M. oryzae* in vivo (Chen *et al*., 2018) and *Xoo* in vitro (Lee *et al*., 2015, 2016). The antimicrobial activity of limonene involves disruption of biofilms integrity, resulting in permeability and disruption of energy metabolism (Han *et al*., 2019; Leite‐Andrade *et al*., 2022; Su *et al*., 2020). According to lab and field studies, rice resistance to sheath blight (Figures 3d, e, 7c, d), bacterial blight (Figure 3g, h), rice blast (Figure 7e–g) was enhanced by (*S*)‐limonene. Due to the resistance of linalool to pathogenic microorganisms (Taniguchi *et al*., 2014; Wang *et al*., 2023), only TPS19‐OE‐9 lines performed more substantial decline rates in lesion length induced by *Xoo* than TPS20‐KO lines. In the field, we observed identical heights across different genotypes (Figure S9b), but lower final panicle number (Figure S9c) and seed weight (Figure S9d) in knockout lines. No increase in panicle number or seed weight was observed in limonene‐overexpressing plants compared with wild‐type (Figure S7c, d), despite limonene may affect plant growth and development through unknown mechanisms. Future work demands broader spatiotemporal scales and more precise yield experiments to observe the effect on growth, development, yield, and quality of rice with enhanced (*S*)‐limonene, particularly in cases of severe disease outbreaks.

**Figure 8:**
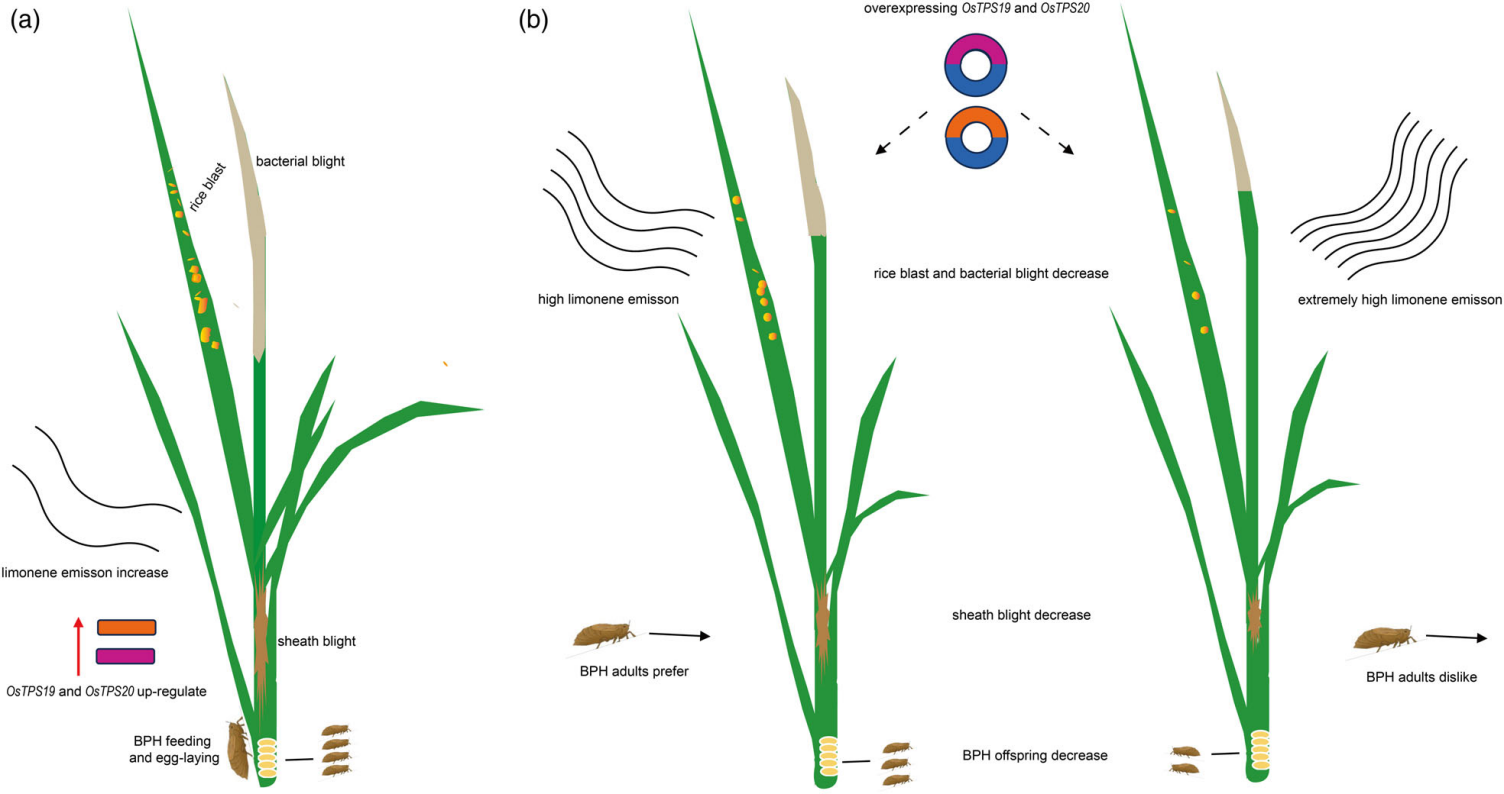
Overexpressing *OsTPS19* and *OsTPS20* increased BPH resistance and various phytopathogen resistance. (a) Model of ZH11. During the growth and development, rice suffered various herbivores (e.g., BPH feeding and egg‐laying) and diseases (e.g., rice blast, sheath blight and bacterial blight), (*S*)‐Limonene synthases (OsTPS19 and OsTPS20) and (*S*)‐limonene were induced by BPH attacking. (b) Model of overexpression rice materials. Overexpression lines of *OsTPS19* and *OsTPS20* emitted more amount of (*S*)‐limonene, suppressed the hatching rate of BPH eggs, performed higher resistance to rice blast, sheath blight, and bacterial blight. BPH showed preference behaviour to overexpression rice materials that emitted higher (*S*)‐limonene, while showing avoidance behaviour to overexpression rice materials that emitted extremely high (*S*)‐limonene.

In this article, we demonstrated that (*S*)‐limonene could serve as a chemical cue to influence host preference of BPH (Figures 3 and 4), and inhibit the BPH egg‐hatching rates for direct plant defence (Figure 5). These findings contribute to the comprehension of volatile‐mediated plant‐herbivores interactions, and provide valuable insights into the potential use of limonene in rice at high concentrations to repel BPH or as a bait plants to attract BPH adult females to reduce the number of subsequent generations. (*S*)‐limonene simultaneously reduced the number of BPH and enhanced the resistance to various important rice diseases in the field (Figure 7), while preventing rice yield loss (Figure S9d). These results provide an environmentally benign approach to improving resistance to both BPH and diseases by manipulating volatile emissions (Figure 8).

## Materials and methods

### Plant and insect cultures

The rice (*Oryza sativa*) genotypes used in this study were wild‐type (Zhonghua 11, ZH11) and different transgenic lines (TPS19‐KO‐3, TPS19‐KO‐4, TPS19‐OE‐2, TPS19‐OE‐9, TPS20‐KO‐1, TPS20‐KO‐4, TPS20‐OE‐1, and TPS20‐OE‐4). Ten days after pre‐germination, rice was planted in soil using three distinct methods: (i) a single plant was planted in a clay pot (12 cm in diameter and 10 cm in height), (ii) a pair of plants (one ZH11 and one transgenic rice) was planted in a clay pot with the same dimensions as above, and (iii) thirty plants were planted in a box (40 cm in length and 30 cm in width), with six plants of each genotype (ZH11, two deficient lines and two overexpression lines of the same gene). Rice was cultivated in the greenhouse at a temperature of 28 ± 4 °C, with relative humidity (RH) of 65 ± 5%, and a photoperiod of 14/10 h (light/dark). Rice plants at the tillering stage (45–50 days old) were used in the experiments unless special instructions were required.

The initial culture of BPH was collected from rice fields in Wuhan, China, and continuously reared on the rice variety TN1 in the greenhouse under 28 ± 2 °C, 75 ± 5% RH and 16:8 h (light/dark) photoperiod.

### Real‐time quantitative PCR

Total RAN was isolated using TRIpure Reagent (Aidlab, China), and 1 mg of total RNA for each sample was subjected to reverse transcription using the PrimeScript RT reagent kit (Takara, Japan). Each qRT‐PCR reaction contained 1 μL of cDNA, 0.5 μL of each primer (10 μM), 10.5 μL of sterilized double distilled H_2_O, and 12.5 μL of SYBR qPCR mix (Aidlab). All reactions were performed on the LightCycler96 (Roche, Switzerland) using a two‐step method, 94 °C for 3 min, 40 cycles of 94 °C for 5 s and 60 °C for 30 s. Relative gene expression levels in different tissues were analysed by the 2^−ΔΔ*Ct*^ method (Schmittgen and Livak, 2008). Three technical replicates were conducted. The *OsUBQ5* gene (AK061988) was used as an internal control. The gene‐specific primers used for qRT‐PCR are provided in Table S1.

### Generation and characterization of transgenic plants

Synthesized PTGs containing two gRNA sequences were inserted into the pRGEB32 transformation vector to generate *OsTPS19* and *OsTPS20* deficient lines, following the same methodology previously described (Xie and Yang, 2013). CDS sequences were cloned from cDNA and subsequently inserted into the pCAMBIA1300 transformation vector, thereby generating *OsTPS19* and *OsTPS20* overexpression lines. The T‐DNA was inserted into the ZH11 through *A. tumefaciens*‐mediated transformation. The resulting homozygous knockout lines were identified by target DNA sequencing. The overexpression lines were screened for a single copy using a Southern blot assay with endonucleases *Hind* III and *Sac* I, as previously described (Southern, 2006). Two T2 homozygous lines of transgenic genotypes were used in subsequent experiments. The list of primers used is presented in Table S1.

### Collection and analysis of plant VOCs

Plants were divided into two groups, non‐infested or infested with 10 gravid BPH female adults for 24 h. The plant VOCs were collected on a daily basis from 8:00 to 18:00 using a closed‐loop dynamic headspace sampling system as previously described by Sun *et al*. (2013). The VOCs from Super Q traps (Alltech Associates, Nicholasville, KY, USA) were eluted by employing 1 mL of n‐hexane (Tedia, Fairfield, OH, USA). Each sample was supplemented with 10 μL of 0.5 mg mL^−1^ nonyl acetate (Sigma‐Aldrich, St. Louis, MO, USA), which served as the internal standard. VOC identification was conducted following the approach outlined by the same method previously described (Sun *et al*., 2020).

To establish a standard curve for (*S*)‐limonene concentration and peak area, (*S*)‐limonene was diluted to 0.5, 1, 5, and 10 μg mL^−1^ with n‐hexane, and samples subsequently analysed by GC–MS. To compare the release of limonene in overexpression lines and knockout lines between different genes, change rates (%) were calculated as follows: (value of transgenic lines – average value of ZH11) × 100/average value of ZH11. Negative values represented a decrease, while positive values represented an increase.

### Preference and performance of BPH

To investigate the colonization and oviposition preference of BPH, 20 gravid BPH female adults were released into a pot with a pair of plants, as previously mentioned (Ye *et al*., 2020). A plastic tube (4 cm in diameter, 15 cm in height, 48 uniformly distributed holes 0.7 mm in diameter on a glass cylinder wall), was positioned to cover the plant stem, with its top end covered with a piece of sponge. The number of BPH on each plant was counted at 1, 2, 4, 8, 12, 24, and 48 h, then BPH was removed, and the number of eggs on each plant was counted under a microscope.

To investigate the host selection preference of BPH, two single‐planted rice plants (one ZH11 and one transgenic) were introduced to the H‐tube olfactometer, respectively. The H‐tube olfactometer was of a similar design to that previously described research by Sun *et al*. (2016). Twenty gravid female adults were placed into the H‐tube olfactometer. The number of BPH within a 5 cm distance from both ends of the transverse tube was counted after 1 h.

To assess the developmental influence of BPH on ZH11 and transgenic rice, 20 newly emerged BPH nymphs were released on plants covered with a plastic tube, as described above. The daily monitoring of BPH and newly emerged adults was conducted on the same date and was transferred to the same new transgenic lines. The number of adults released on each plant was <5 pairs, and adults that died within the first 2 days were excluded from the study. The number of BPH nymphs that metamorphosed into adults, the weight of the resulting adults, the survival rates of the BPH nymphs, and the survival days of the adults were recorded.

To determine the hatching rate of BPH eggs deposited on ZH11 and transgenic plants, two pairs of newly emerged female and male adults were introduced to rice stems that were covered with a plastic tube, as mentioned above for 10 days. The number of newly hatched nymphs was counted daily until no further nymph emergence was observed. The unhatched eggs from the oviposition sheath were counted using a microscope.

To compare the plant tolerance to BPH attack between ZH11 and transgenic plants, 450 gravid BPH female adults were released into a box with rice plants and wrapped with a gauze cage (60 cm in length × 60 cm in width × 70 cm in height). The plants were examined daily, and the death rate of plant leaves (%), number of dead leaves × 100/total number of leaves was recorded and photographed after 18 days. Considering the interactions between different genotypes, the change rates (%) of the overexpression lines were calculated as follows: (value of transgenic lines – average value of ZH11 and knockout lines) × 100/average value of ZH11 and knockout lines.

### Pathogen inoculation

The *Rhizoctonia solani strain* WH‐1 was originally isolated from a rice paddy in Wuhan, China, and cultured in a potato sucrose agar medium at 26 °C. Mycelial blocks (1 cm in diameter) at the growth edge were introduced to the sheath, covered with wet sterile silver paper and then cultivated on plants using the methods described above. The lesion length was measured and photographed at 7 days post‐incubation (dpi).

The *Xanthomonas oryzae* pv. *oryzae* strain XG‐25 (race 4) was isolated from a rice paddy in Wuhan, China, and cultured on potato sucrose agar medium at 26 °C. A single colony was inoculated in a liquid lysogeny broth medium and incubated at 220 rpm for 10 h at 37 °C. The bacterial suspension was diluted to approximately 10^9^ colony‐forming units (CFU) mL^−1^ using sterile distilled water. The scissors were immersed in the bacterial suspension and used to cut 2 cm of rice plant leaves. After inoculation, the rice plants were maintained in a controlled climate box (30 ± 1 °C, 90 ± 5% RH, and 20 000 lx light intensity) for 12 h. Subsequently, the plants were transferred to the greenhouse and grown under the same conditions. The lesion length was then measured and photographed at 14 dpi.

To compare the lesion length between OsTPS19 overexpression lines and OsTPS20 overexpression lines, the change rates (%) were calculated as follows: (value of transgenic lines – average value of ZH11) × 100/average value of ZH11.

### Synthetic standard (*S*)‐limonene assays

H‐tube olfactometer experiments were carried out to investigate the influence of (*S*)‐limonene on BPH host performances. The rubber lures were soaked in solutions of synthetic (*S*)‐limonene (Sigma‐Aldrich, St. Louis, MO, USA) at concentrations of 0.01, 0.1, 1, 2, 4, 6, 8, and 10 mg mL^−1^, which were diluted with methanol (Tedia, Fairfield, OH, USA) for 2 days. The wild‐type ZH11 add (*S*)‐limonene rubber lures were placed in one arm as odorant sources, while the wild‐type ZH11 add methanol rubber lures were placed in the other arm as a control. Twenty gravid female adults were introduced into the transverse tube. The number of BPH within a 5 cm distance from both ends of the transverse tube was counted after 1 h. To ascertain the actual release rate of (*S*)‐limonene, the volatile compounds released from the rubber lures were collected and analysed using a comparable methodology to that described above. The collection of (*S*)‐limonene was performed nine times for each concentration, and three samples of the same concentration were mixed into one sample and analysed by GC–MS.

To investigate the effect of (*S*)‐limonene on the hatching rate of BPH eggs, (*S*)‐limonene was diluted with lanolin (Sigma‐Aldrich, St. Louis, MO, USA) to ensure the continuous existence of (*S*)‐limonene. Fresh and intact BPH eggs laid within 12 h were collected from the rice leaf sheath. Every 100 BPH eggs were covered by sterile paper (2 cm in semidiameter). The paper was pretreated with 200 μL of 0, 0.001, 0.01, 0.1, 1 and 10 mg mL^−1^ (*S*)‐limonene and H_2_O, respectively. The BPH eggs were incubated in a culture dish (10 cm in diameter, 1.5 cm in height) within a controlled climate box (28 ± 1 °C, 75 ± 5% RH and 16:8 h (light/dark) photoperiod). The number of hatched BPH nymphs was recorded until no nymphs emerged. In another experiment, sheaths were daubed with 200 μL of (*S*)‐limonene at concentrations of 0, 0.01, and 1 mg mL^−1^, respectively. After 2 h, 10 gravid BPH female adults were introduced to the sheaths for 24 h and removed. The number of newly hatched nymphs was counted daily until no nymphs emerged. The number of unhatched eggs from the deposited sheath was counted under a microscope.

To evaluate the impact of (*S*)‐limonene on adjacent plants, rubber lures soaked in solutions containing 0, 0.01, and 1 mg mL^−1^ (*S*)‐limonene for 2 days were placed 5 cm from the rice plants. One day later, ten gravid BPH female adults were introduced to the sheath for 24 h and removed. As previously stated, the number of newly hatched nymphs and unhatched eggs from the leaf sheath were counted.

### Field experiments

The field experiments were conducted in Wuhan, Hubei Province, China (114.30° E, 30.60° N) in 2023. Seedings of ZH11 plants and transgenic lines were germinated and cultivated in the nursery field. After 30 days, the rice plants were transferred into the field blocks. Each experimental block (2 × 2 m) contained 100 hills of plants and was surrounded by a 0.5 m ZH11 buffer zone. Each line was randomly assigned three blocks. Five tillers of 20 plants from each block were investigated at 30, 40, 50, 60, and 70 days after planting to count the total numbers of all BPHs. A total of 30 plants were randomly selected from each block to record the number of tillers, tillering leaves, and leaves damaged by the RLR. The damage rate (%) of the RLR was calculated as the total number of damaged leaves × 100/(average tiller number per plant × average leaf number per tiller), at 50 and 70 days. The whole block was examined to record the number of tillers damaged by SSB at 50 and 70 days. Five tillers from 30 plants were randomly selected from each block to calculate the disease index (DI) for bacterial blight, rice sheath blight, and rice leaf blast at 50 and 70 days, and rice panicle blast at 80 days. DI was calculated using the formula described by Raupach *et al*. (1996): DI = [∑ (rating number × number of plants in rating) × 100%]/(total number of plants × highest rating). The disease level is classified according to the area of the lesion with levels ranging from 0 (no lesion), 1 (lesion area <1%), 3 (lesion area <5%), 5 (lesion area <25%), 7 (lesion area <50%) and 9 levels (lesion area more than 50%). Twenty plants per block were randomly harvested to measure the number of panicles per plant, 15 plants for the height per plant, and five tillers of 10 plants for the seed yield.

### Data analysis

The study analysed variations in volatile compounds emission, herbivore performance, disease lesion length, egg‐hatching rate following (*S*)‐limonene pretreatment, pest population dynamics, DIs, and yield traits were determined by ordinary one‐way analysis of variance (ANOVA) with Tukey's HSD test. The homogeneity of variance and normality of distribution in the datasets were determined by the application of Leneve's test and the Shapiro–Wilk test, respectively. Log transformation was applied to datasets that did not follow a normal distribution or exhibited unequal variance. The impact of BPH infestation on rice performance was evaluated using the Kruskal–Wallis test with Dunn's test, given the non‐normally distributed log‐transformed datasets. The differences in the H‐tube olfactometer bioassay were determined using the χ‐squared test. The variation between the two experimental treatments was evaluated using the Student's *t*‐test. All tests were carried out using R (v.4.3.0, R project) and GraphPad Prism (v.9.2.0, GraphPad Software). Comprehensive statistical analysis values for all experiments are provided in Data S1.

## Author contributions

CLQ performed most experiments, analysed data, and wrote the manuscript; WL, LNW, and SCW participated in the genetic transformation and identification of genetically modified rice; SF, CW, and SYY participated in the field experiments; STA and JL revised the manuscript. YJL provided critical suggestions for experiments; MQW provided critical suggestions for experiment design and revised the manuscript.

## Conflict of interest

The authors declare no competing financial interest.

## Supporting information


**Figure S1** Standard curve of (*S*)‐limonene concentration and peak area.
**Figure S2** BPH infestation affected the relative amounts of volatiles from ZH11.
**Figure S3** *OsTPS19* and *OsTPS20* had different transcript expression profiles in ZH11.
**Figure S4** Sequencing peak plot about knockout lines of *OsTPS19* and *OsTPS20*.
**Figure S5** Southern blot analyses of overexpression lines of *OsTPS19* and *OsTPS20*.
**Figure S6** *OsTPS19* and *OsTPS20* slightly affected other volatiles emitted from transgenic rice.
**Figure S7** *OsTPS19* and *OsTPS20* did not affect development of BPH.
**Figure S8** *OsTPS19* and *OsTPS20* affected damage of SSB but not affected RLR.
**Figure S9** Knocking out *OsTPS19* and *OsTPS20* had fewer panicles and lighter seeds.
**Data S1** Comprehensive statistical analysis values of figures.

**Table S1** Primers used for PCR and qRT‐PCR.

## Data Availability

The data supporting the findings of this investigation are accessible in the supplemental material of this publication.
