# Supplementary material for: Limonene enhances rice plant resistance to a piercing‐sucking herbivore and rice pathogens
Source: Plant Biotechnol J. 2024 Sep 28;23(1):84–96. doi: 10.1111/pbi.14481 (PMC11672756; doi:10.1111/pbi.14481)
Supplement: Supplementary file 1 — Figure S1 Standard curve of (S)‐limonene concentration and peak area. Figure S2 BPH infestation affected the relative amounts of volatiles from ZH11. Figure S3 OsTPS19 and OsTPS20 had different transcript expression profiles in ZH11. Figure S4 Sequencing peak plot about knockout lines of OsTPS19 and OsTPS20. Figure S5 Southern blot analyses of overexpression lines of OsTPS19 and OsTPS20. Figure S6 OsTPS19 and OsTPS20 slightly affected other volatiles emitted from transgenic rice. Figure S7 OsTPS19 and OsTPS20 did not affect development of BPH. Figure S8 OsTPS19 and OsTPS20 affected damage of SSB but not affected RLR. Figure S9 Knocking out OsTPS19 and OsTPS20 had fewer panicles and lighter seeds. Data S1 Comprehensive statistical analysis values of figures. [file PBI-23-84-s002.docx]

**Figure** **S1** Standard curve of (*S*)-limonene concentration and peak area. Values presented were means.


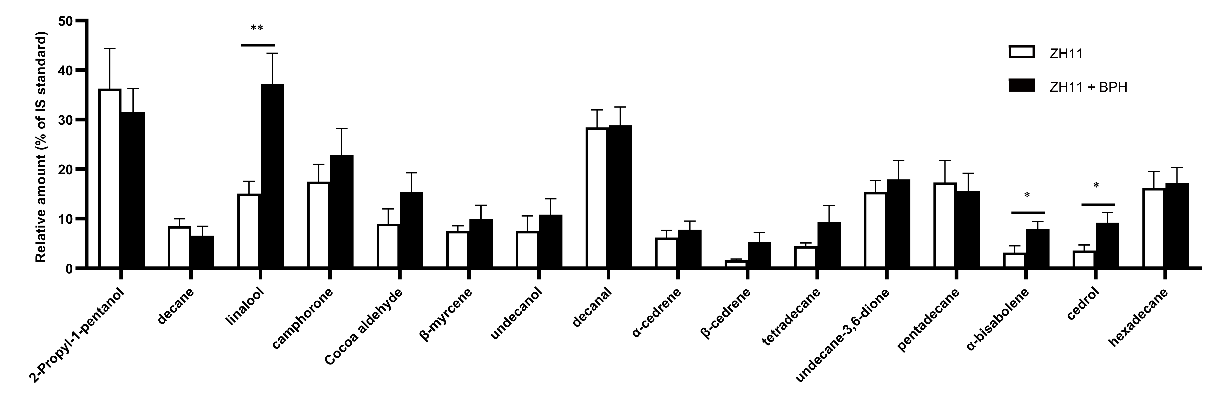


**Figure** **S2** BPH infestation affected the relative amounts of volatiles from ZH11. ZH11: ZH11 non-infested with BPH, ZH11 + BPH: ZH11 infested with 10 gravid BPH female adults for 24 hours. Values presented are the means ± SE, n = 6. Asterisks indicate statistically significant differences (**P* < 0.05, ***P* < 0.01), Student's *t*-tests.


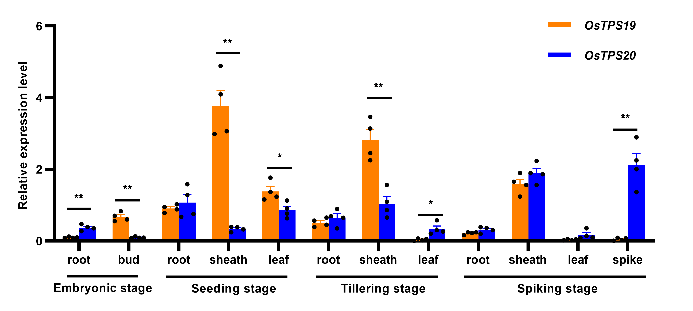


**Figure** **S3** *OsTPS19* and *OsTPS20* had different transcription expression profiles in ZH11. The experiments were repeat four times. Embryonic stage: 2 days after pre-germination, seeding stage: 20 days after planting, tillering stage: 40 days after planting, tillering stage, spiking stage: 80 days after planting. Values presented are the means ± SE. Asterisks indicate statistically significant differences, Student's *t*-tests, **P* < 0.05, ***P* < 0.01.


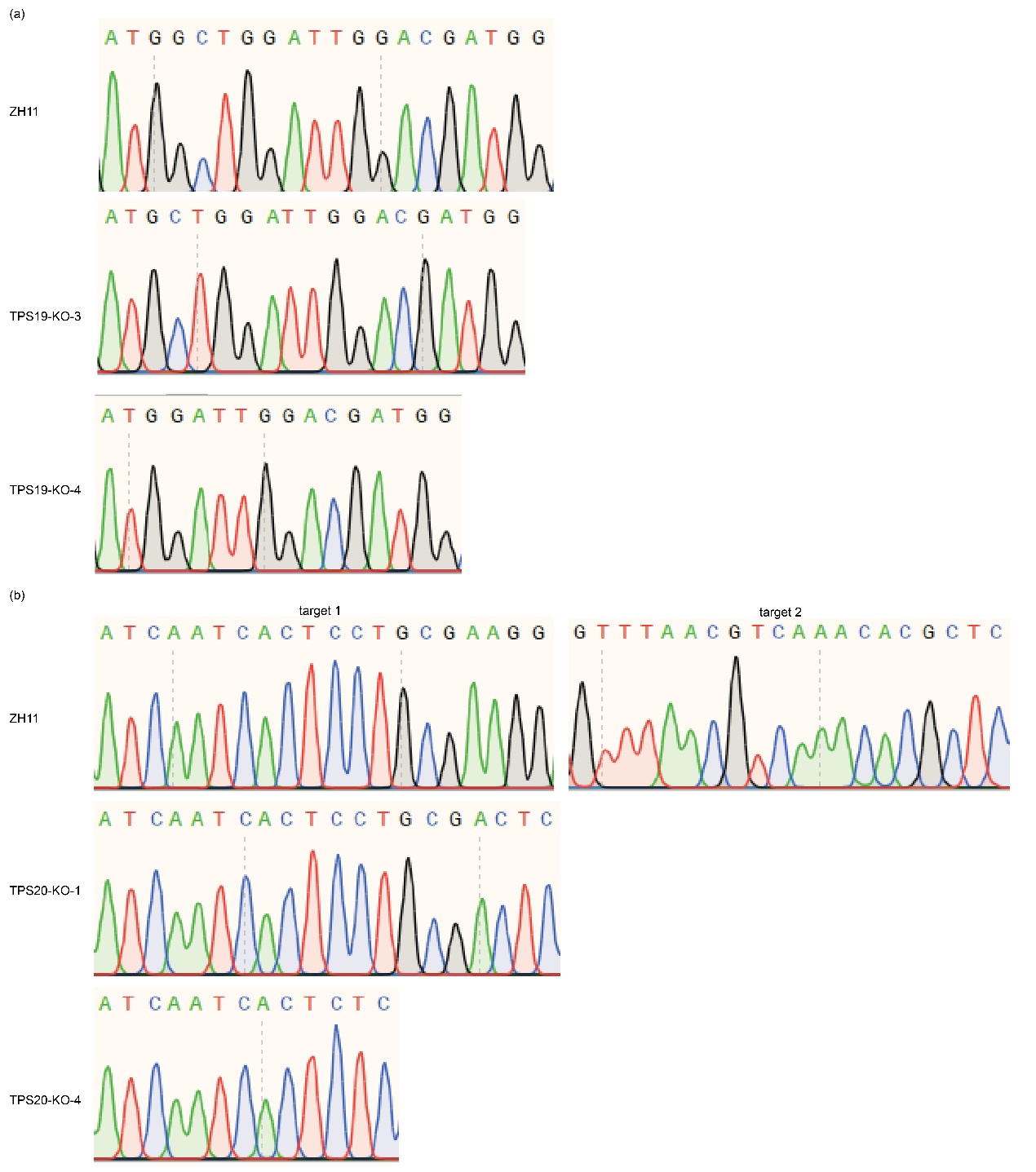


**Figure** **S4** Sequencing peak plot about knockout lines of *OsTPS19* (a) and *OsTPS20* (b)*.*

**
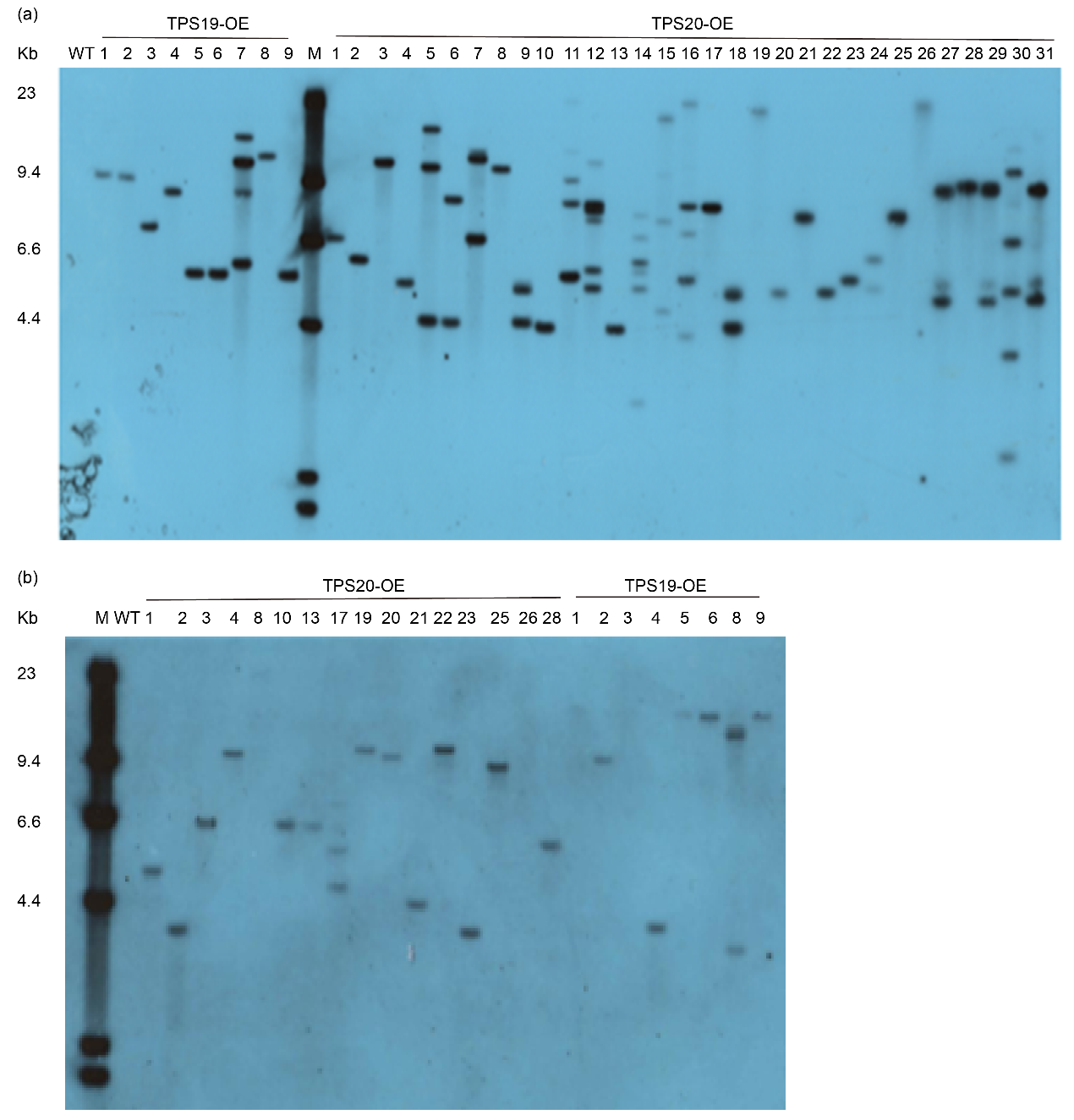
**

**Figure** **S5** Southern blot analyses of overexpression lines of *OsTPS19* and *OsTPS20* that digested with endonuclease *Hin*d III (a) and *Sac* I (b). M: DNA molecular marker, WT: wild-type ZH11.


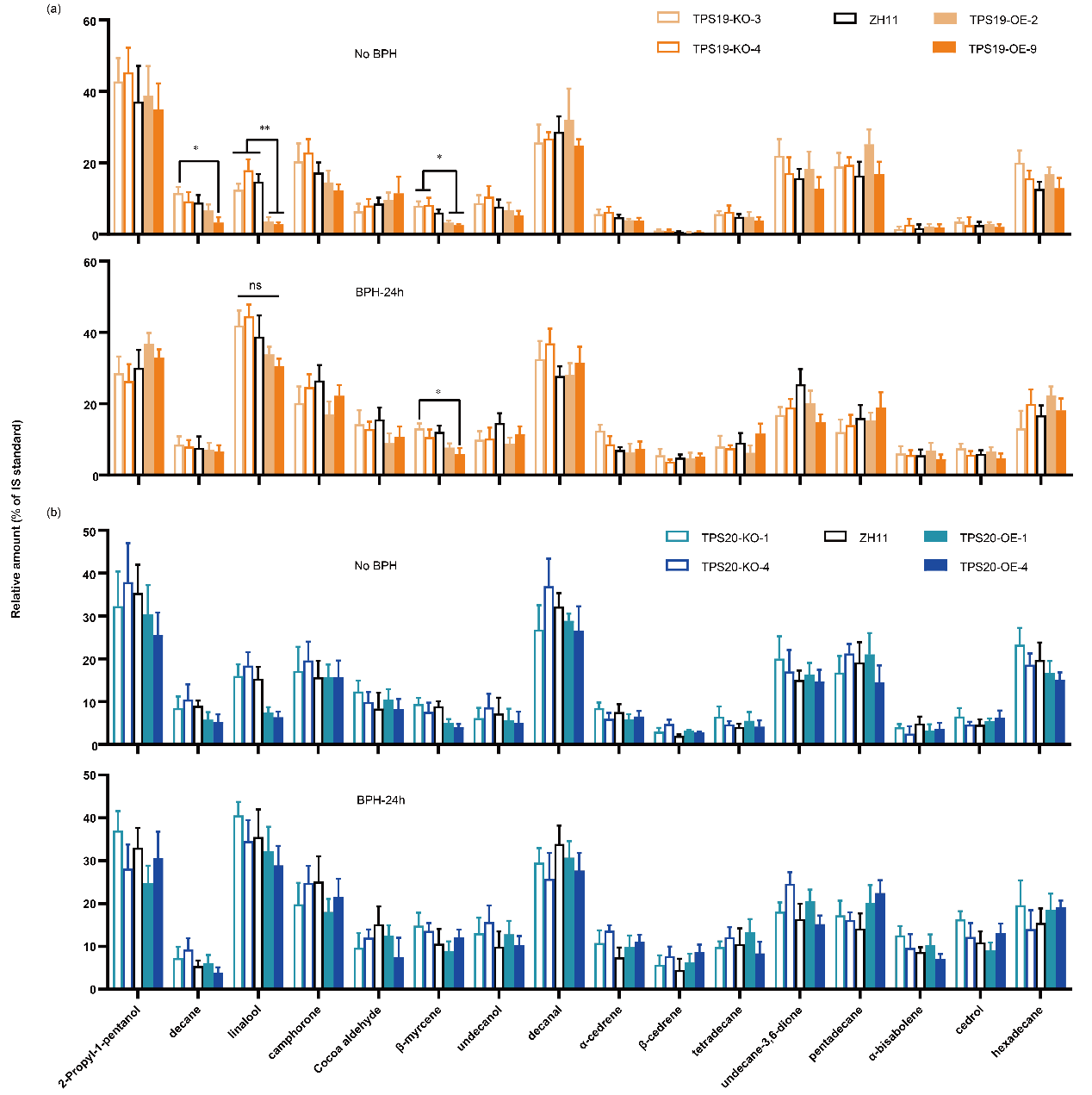


**Figure S6** *OsTPS19* and *OsTPS20* slightly affected other volatiles emission of transgenic rice plants. (a) Volatiles emitted from transgenic plants of *OsTPS19* non-infested or infested with 10 gravid BPH female adults for 24 hours, n = 5. (b) Volatiles emitted from transgenic plants of *OsTPS20* non-infested or infested with 10 gravid BPH female adults for 24 hours, n = 5. Values presented are the means ± SE. Asterisks indicate statistically significant differences (**P* < 0.05, ***P* < 0.01), ordinary one-way ANOVAs with Tukey’s HSD test.


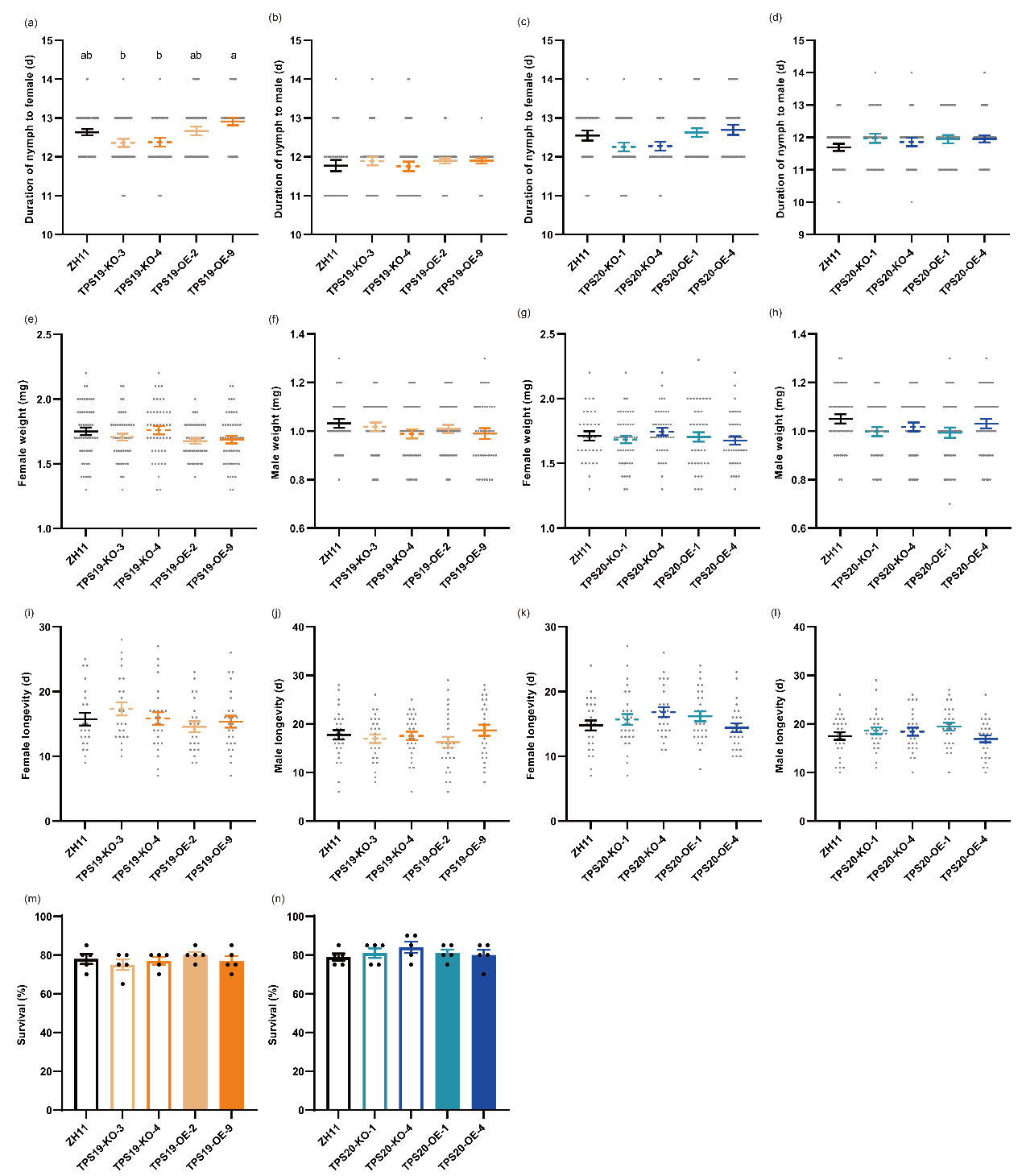


**Figure S7** *OsTPS19* and *OsTPS20* did not affect development of BPH. (a - d) Duration of BPH nymphs to adults on ZH11 or transgenic lines of *OsTPS19* and *OsTPS20*, n = 31 - 49. (e - h) Weight of BPH female adults and male adults on ZH11 or transgenic lines of *OsTPS19* and *OsTPS20*, n = 31 - 49. (i - l) Longevity of BPH female adults and male adults on ZH11 or transgenic lines of *OsTPS19* and *OsTPS20*, n = 23 - 29. (m, n) Survival of BPH from nymphs develop to adults on ZH11 or transgenic lines of *OsTPS19* and *OsTPS20*, n = 5. Values presented are the means ± SE, dots represent biological replicates. Different lowercase letters indicate statistically significant differences (*P* < 0.05), ordinary one-way ANOVAs with Tukey’s HSD test.


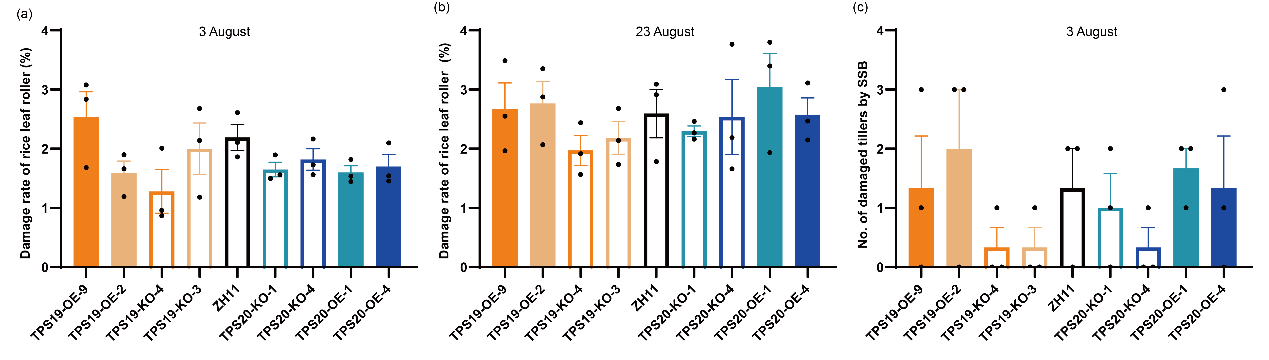


**Figure S8** *OsTPS19* and *OsTPS20* affected damage of SSB but not affected RLR. (a, b) Damage rate of rice leaf roller (%) at 3 August and 23 August, n = 3. (c) Number of tillers damaged by SSB at 3 August and 23 August, n = 3. Values presented are the means ± SE, dots represent biological replicates, ordinary one-way ANOVAs with Tukey’s HSD test were used.

**
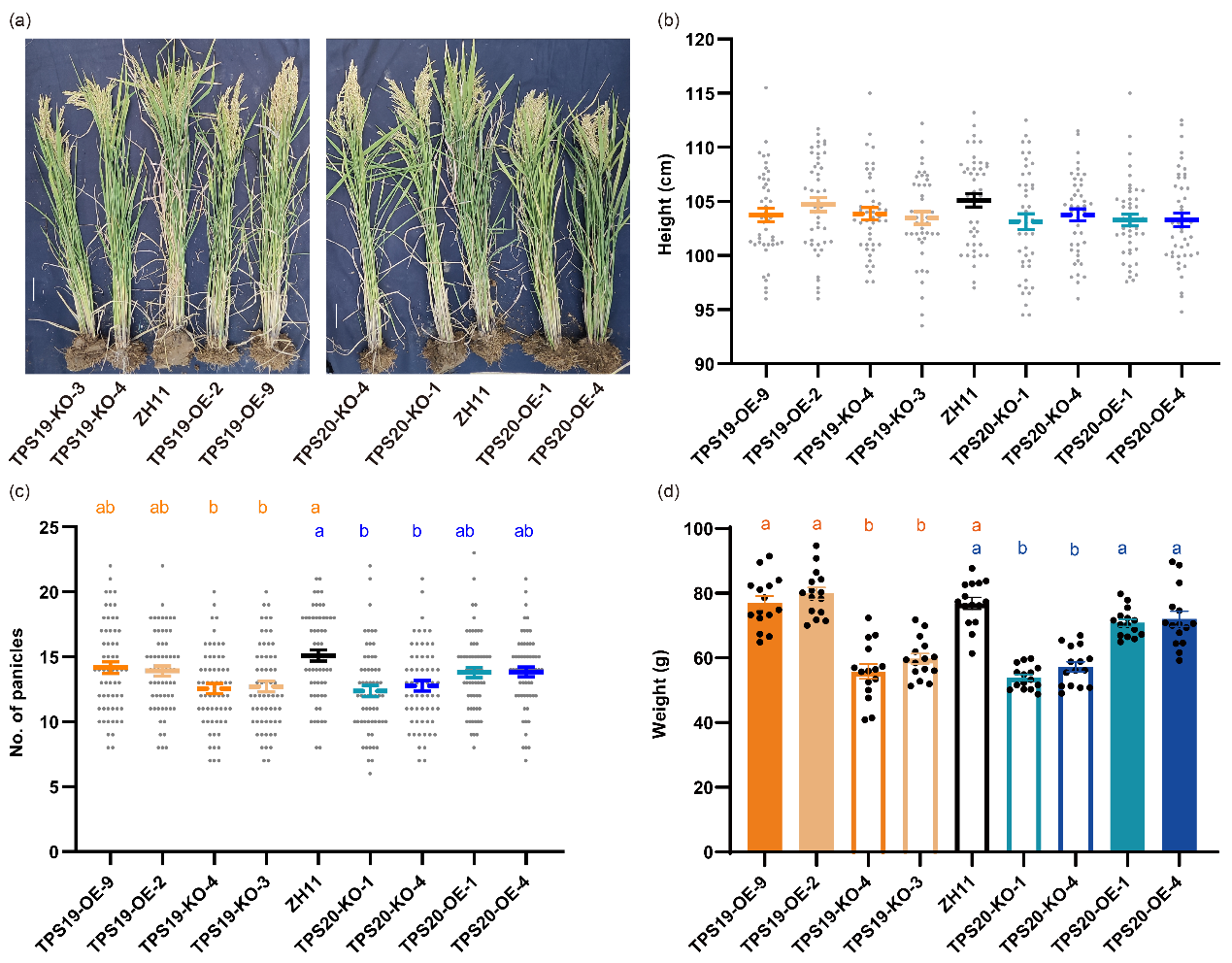
**

**Figure S9** Knocking out *OsTPS19* and *OsTPS20* had fewer panicles and lighter seeds. (a) Phenotypes at harvest, bar indicate 10 cm. (b) Height of per plant at harvest, n = 3 × 15. (c) Number of panicles per plant at harvest, n = 3 × 20. (d) Seed weight of per two plants, n = 3 × 5. Values presented are the means ± SE, dots represent biological replicates. Different orange lowercase letters indicate statistically significant differences (*P* < 0.05) between ZH11 and transgenic lines of *OsTPS19*, different blue lowercase letters indicate statistically significant differences (*P* < 0.05) between ZH11 and transgenic lines of *OsTPS20*, ordinary one-way ANOVAs with Tukey’s HSD test.
