# Supplementary material for: Limonene enhances rice plant resistance to a piercing‐sucking herbivore and rice pathogens
Source: Plant Biotechnol J. 2024 Sep 28;23(1):84–96. doi: 10.1111/pbi.14481 (PMC11672756; doi:10.1111/pbi.14481)
Supplement: Supplementary file 2 — Table S1 Primers used for PCR and qRT‐PCR. [file PBI-23-84-s001.docx]

Table S1 Primers used for PCR and qRT-PCR

| Gene  Name | Description | Forward (F)/  Reverse (R) | Sequences (5’–3’) |
| --- | --- | --- | --- |
| *OsTPS19* | qRT-PCR | F | CCGTAGCAGTGCCATTCTT |
|  |  | *R* | TCCCAACATTTCCCTGATGATAC |
| *OsTPS20* | qRT-PCR | F | GCAACGACTTTTGAACTT |
|  |  | *R* | CCAACAATCTCCTGAATATAC |
| *OsUBQ5* | qRT-PCR | F | AACCACTTCGACCGCCACT |
|  |  | *R* | GTTCGATTTCCTCCTCCTTCC |
| *OsTPS19* | CDS clone | F | ATGTCAACTTCCATCCCTC |
|  |  | *R* | CTAAAGGGTGACAGGATTC |
| *OsTPS20* | CDS clone | F | ATGTCTACTTCCATCCCTC |
|  |  | *R* | CTAGATGGGGACAGGATTCAC |
| *OsTPS19* | Genomic DNA  sequencing | F | AGCAACGATCAGACTACCAC |
|  |  | R | GATCATTCTTTTAGGTGCCC |
| *OsTPS20* | Genomic DNA  sequencing | F | CATGGTGTTGTGCATGCTAG |
|  |  | R | GTTATGCACTCGAGCATCTT |
| *OsSPS* | qRT-PCR | F | TTGCGCCTGAACGGATAT |
|  |  | R | CGGTTGATCTTTTCGGGATG |
|  |  | F | AGGGTTACCGTGGAACACAC |
| EPSPS | qRT-PCR |  |  |
|  |  | R | AACGTCGATGCCGTGAATCT |

1
